# Using Biosensors to Study Protein–Protein Interaction in the Hippo Pathway

**DOI:** 10.3389/fcell.2021.660137

**Published:** 2021-04-26

**Authors:** Alexander Pipchuk, Xiaolong Yang

**Affiliations:** Department of Pathology and Molecular Medicine, Queen’s University, Kingston, ON, Canada

**Keywords:** Hippo pathway, biosensor, luciferase, protein–protein interaction, NanoBiT, NanoLuc

## Abstract

The Hippo signaling network is dependent on protein–protein interactions (PPIs) as a mechanism of signal transduction to regulate organ size, cellular proliferation and differentiation, tumorigenesis, and other cellular processes. Current efforts aim to resolve the complex regulation of upstream Hippo components or focus on identifying targeted drugs for use in cancer therapy. Despite extensive characterization of the Hippo pathway interactome by affinity purification mass spectrometry (AP-MS) and other methodologies, previous research methods have not been sufficient to achieve these aims. In this review, we describe several recent studies that make use of luciferase-based biosensors as a new approach to study the Hippo Pathway. These biosensors serve as powerful tools with which to study PPIs both *in vitro* using purified biosensor proteins, and in real time in live cells. Notably, luciferase biosensors have excellent sensitivity and have been used to screen for upstream kinase regulators of the Hippo pathway. Furthermore, the high sensitivity and stability of these biosensors enables their application in high throughput screening for Hippo-targeted chemotherapeutics. Finally, we describe the strengths and weaknesses of this method relative to AP-MS and discuss potential future directions for using biosensors to study Hippo signaling.

## Introduction

### The Hippo Pathway

The Hippo pathway is an evolutionarily conserved signaling cascade that plays central roles in human physiology and disease (Pan, 2010; Ma et al., 2019). This signaling pathway has been connected to a wide variety of processes, including development (e.g., organ size control, early embryogenesis, skeletal development) (Kegelman et al., 2020; Wu and Guan, 2021), tissue homeostasis (regeneration, fibrosis, cell proliferation, cell death, differentiation, and stem cell renewal) (Kim et al., 2019; Cao et al., 2020; Dey et al., 2020), mechanotransduction (Meng et al., 2016; Misra and Irvine, 2018; Ma et al., 2019; Zhang et al., 2020), cardiovascular development and disorders (cardiomyocyte proliferation and cardiac injury) (Moya and Halder, 2019; Chen et al., 2020), diabetes (e.g., insulin/glucose metabolism, β-cell function) (Ardestani and Maedler, 2018; Ardestani et al., 2018), neurodegenerative disease (neuronal apoptosis) (Sahu and Mondal, 2020a, b), and cancer progression and therapy (tumorigenesis, angiogenesis, metastasis, immune response, and drug resistance) (Visser and Yang, 2010; Lai et al., 2012; Zhao and Yang, 2015, 2019; Janse van Rensburg and Yang, 2016; Yeung et al., 2016; Taha et al., 2018; Wu and Yang, 2018; Azad et al., 2019).

**FIGURE 1:**
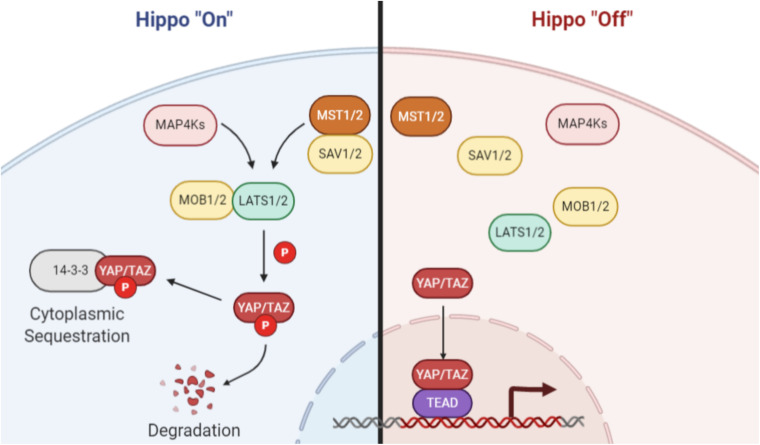
Canonical Hippo signaling. When Hippo signaling is activated, MAP4 kinases or the MST1/2 kinases phosphorylate and activate the LATS family of kinases. The MST/LATS interaction is facilitated by MOB and SAV adaptor proteins. Activated LATS kinases subsequently phosphorylate the YAP and TAZ paralogs, which then associate with 14-3-3 proteins and are sequestered in the cytoplasm or degraded. When Hippo signaling is inactive, YAP/TAZ are not phosphorylated and are free to translocate to the nucleus, where they act as coactivators of transcription through interaction with TEAD family and other transcription factors. Image created with BioRender.com.

The canonical Hippo pathway (Figure 1), first identified in *Drosophila* and later in mammals (Xu et al., 1995; St John et al., 1999), is depicted as two kinases that act in series to regulate downstream co-activators of transcription. In mammals, upstream regulators activate the MST1/2 kinases, which in turn mediate phosphorylation of the LATS family of kinases (Chan et al., 2005). The MST1/2-LATS interaction is facilitated by the SAV1 and MOB1 adaptor proteins (Bae and Luo, 2018). Phosphorylation of LATS results in its activation and subsequent phosphorylation of two paralogous transcriptional coactivators: YAP and TAZ (YAP/TAZ). Phosphorylated YAP/TAZ is sequestered in the cytoplasm and is unable to associate with the TEAD family of transcription factors in the nucleus to direct transcription of Hippo-associated genes. Notably, the YAP/TAZ-TEAD interaction has been identified as a promising drug target for molecular therapeutics (Liu-Chittenden et al., 2012; Johnson and Halder, 2014; Zhou et al., 2015; Wu and Yang, 2018).

Regulation of the Hippo pathway extends far beyond the canonical kinase cascade (Yu and Guan, 2013). Several MAP4K family proteins (Meng et al., 2015), NF2/merlin (Hamaratoglu et al., 2006; Zhang et al., 2010; Yin et al., 2013), angiomotin (Zhao et al., 2011; Li et al., 2015), kibra (Yu et al., 2010), and expanded (Hamaratoglu et al., 2006) have also been established as influential regulators of Hippo signaling. Adding further complexity, the Hippo pathway is engaged in crosstalk with other signaling cascades at the level of YAP and TAZ (Piccolo et al., 2014; Totaro et al., 2018; Zhao et al., 2018), and several mass spectrometry analyses have revealed an extensive Hippo pathway interactome (Couzens et al., 2013; Kwon et al., 2013; Wang et al., 2014). Overall, the large network of dynamic protein–protein interactions (PPIs) in the Hippo pathway has made it difficult to study using traditional techniques.

### Luciferase Biosensors

Bioluminescence is the process whereby light is produced and emitted by a living organism. This phenomenon, which is distinct from fluorescence, famously occurs in fireflies and many species of marine animals. At the chemical level, the light-emitting reaction is catalyzed by a luciferase enzyme in the presence of its substrate, a luciferin. The light produced can be easily measured by a photometer, allowing for non-invasive, real time monitoring of cellular processes. As such, luciferase-based technologies hold a broad range of utility in cancer and molecular biology research, with applications for use as a reporter of gene expression, a marker of cellular proliferation, *in vivo* tumor imaging, and more (Badr and Tannous, 2011; Xu et al., 2016).

**FIGURE 2:**
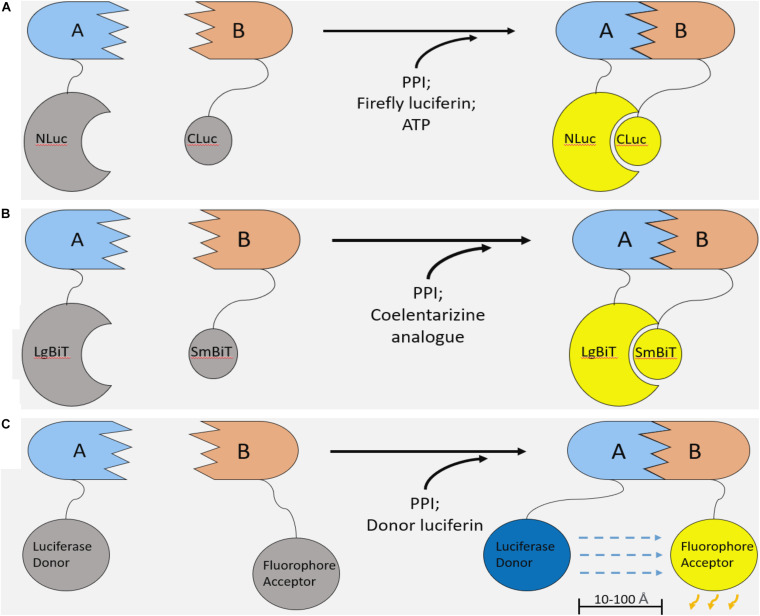
Luciferase-based methods of studying protein–protein interaction. **(A)** Firefly Luciferase complementation system. Two components of the firefly luciferase are fused with two interacting proteins. Upon PPI between the fusion proteins, luciferase complementation occurs, and, in the presence of a luciferin substrate and ATP, light is emitted. **(B)** NanoBiT system. The NanoBiT system is a split luciferase technology derived from a deep-sea shrimp. It is comprised of the 18kDa LgBiT and 1.3 kDa SmBiT. The main advantages of NanoBiT over other split luciferase systems are its small size, high sensitivity, and improved thermal stability. **(C)** Bioluminescence resonance energy transfer (BRET). Like split luciferase complementation assays, BRET is another mechanism of quantifying PPIs in real time. A functional luciferase donor and fluorophore acceptor are fused onto an interacting pair of proteins. Upon PPI, the fluorophore and luciferase are brought into proximity (10–100 Å). If the conditions are met for the luciferase to emit light (i.e., the presence of a luciferin and any necessary cofactors), the bioluminescent light will excite the fluorophore, which can emit light of a different wavelength.

Additionally, split luciferase systems allow for the development of complementarity assays to measure protein-protein interactions (Figures 2A,B; Azad et al., 2014). In principle, a luciferase enzyme is divided into two component parts, abolishing its activity. Each of the two luciferase constituents can be fused onto two interacting proteins. Upon PPI between these recombinant proteins, the luciferase components reform a functional enzyme capable of emitting light in the presence of its substrate (Figures 2A,B). Therefore, these systems enable non-invasive, real time monitoring of PPIs and provide several key advantages over previous methods of PPI detection.

Our lab has recently developed several split luciferase systems to study PPIs within the Hippo pathway. Using the same luciferase-based technology, another group has developed a separate Hippo pathway biosensor platform that differs mechanistically from our system but may be used for many of the same purposes. In this review, we describe how these systems were designed and what they have since been used to accomplish. We discuss the advantages and disadvantages of these systems over other methods of studying PPIs, and, importantly, explore future directions for the application of this technology to study Hippo signaling.

## Body

### Firefly Luciferase-Based LATS Biosensor

Historically, split luciferase biosensors have been derived from enzymes present in firefly or renilla species. In 2018, we presented research that made use of a firefly luciferase (FLuc)-based LATS biosensor to identify VEGFR as an upstream regulator of Hippo signaling (Azad et al., 2018). For this work, an N terminal segment of FLuc (called NLuc, consisting of amino acids 1-416) was fused with a 15 amino acid segment of the YAP protein (YAP15, consisting of residues 120-134). YAP15 contains the consensus LATS substrate identification sequence (HxH/R/KxxS/T; H-histidine; R-arginine; K-lysine; S-serine; T-threonine) and a critical LATS1/2 kinase phosphorylation site, S127 (Zhao et al., 2007; Hao et al., 2008). Upon S127 phosphorylation by LATS, YAP associates with 14-3-3 proteins in the cytoplasm and is prevented from binding to nuclear transcription factors (Zhao et al., 2007). As such, the C-terminal segment of FLuc (CLuc) was cloned onto 14-3-3.

Upon phosphorylation of NLuc-YAP15 (“A”) and association with CLuc-14-3-3 (“B”), the luciferase constituents reform a functional enzyme that emits light in the presence of the luciferin substrate (Figure 2A). The emitted light intensity can be measured by a luminometer to quantify protein–protein interaction between YAP15 and 14-3-3. Thus, the NLuc-YAP15 and CLuc-14-3-3 fusion proteins, when co-expressed, constitute a biosensor for LATS activity. This LATS biosensor responded as expected to the overexpression or inhibition of upstream Hippo pathway components, showing increased bioluminescent activity when co-transfected with MST, and decreased luminescent activity upon inhibition of the Hippo pathway. The LATS biosensor was further validated by mutating 3 distinct residues on the consensus LATS binding motif (H, R, and S), all of which abolished luminescent activity and S127 phosphorylation. Significantly, the LATS biosensor was also used to monitor LATS activity in vivo in a xenograft tumor mouse model. Overall, the validation procedures convincingly established the LATS biosensor as an accurate method to assess LATS kinase activity both in vitro and in vivo through quantitation of YAP and 14-3-3 interaction.

Furthermore, the FLuc LATS biosensor was used to screen for upstream kinase regulators of the Hippo pathway (Azad et al., 2018). In this approach, the LATS biosensor components were transfected into a cell line that was subsequently exposed to 80 distinct kinase inhibitors. The results of the screen revealed 6 kinase inhibitors that activated the biosensor, some of which targeted kinases that had previously been established as Hippo pathway regulators. Ultimately, VEGFR was identified as a novel upstream LATS regulator. Mainly, this study demonstrates the application of split luciferase systems as a powerful tool to study complex signaling networks and facilitate the discovery of novel regulators.

### Improving the LATS Biosensor Using NanoLuc Binary Technology

We improved upon the LATS biosensor in a subsequent iteration using NanoLuc binary technology (NanoBiT). NanoLuc luciferase is derived from the deep-sea shrimp *Oplophorus gracilirostris* and holds several advantages over firefly or renilla based technologies (Hall et al., 2012; England et al., 2016). NanoLuc is roughly three-fold smaller than firefly luciferase, potentially limiting steric inhibition of PPIs in the context of a split luciferase assay. Also, this luciferase is ATP-independent and, importantly, shows improved thermal stability at 37°C. Finally, NanoLuc is over 100-fold brighter than other luciferase enzymes, enabling the development of more sensitive assays. In the NanoBiT system, NanoLuc luciferase is split into two components: the 18 kDa Large BiT (LgBiT) and the 1.3 kDa, 11 amino acid Small BiT (SmBiT; Figure 2B). Similar to other split luciferase systems (Figure 2A), these components have been extensively engineered such that their association is dictated by the interaction of the target proteins to which they are attached (Dixon et al., 2016). In other words, the NanoBiT constituents SmBiT and LgBiT do not associate unless they are brought into proximity of each other by PPI between the target proteins. This allows for accurate and sensitive measurement without drastically altering the dynamics of a given PPI.

To improve upon the FLuc-LATS biosensor, the LgBiT constituent was fused with YAP15 (Protein “A”), and SmBiT was attached to 14-3-3 (Protein “B”, Figure 2B; Nouri et al., 2019a). This NLuc-LATS biosensor indeed showed ∼150-fold increased luminescent intensity and improved thermal stability when compared to the FLuc-Lats biosensor. Following validation, the NLuc-LATS biosensor was then used to conduct an expanded, kinome wide high throughput screen for upstream kinase regulators. Of 560 compounds screened, 54 kinase inhibitors increased the bioluminescent signal more than two-fold compared to controls treated with DMSO. Like the FLuc-LATS biosensor screen, many of these compounds targeted kinases that had previously been established as Hippo pathway regulators, including EGFR (Fan et al., 2013), PI3K (Fan et al., 2013; Zhao et al., 2018), MAPK (Meng et al., 2015), and VEGFR inhibitors (repeating the findings from the FLuc-LATS biosensor screen). Ultimately, anaplastic lymphoma kinase (ALK) was identified and validated as a novel Hippo pathway regulator acting through LATS to affect YAP/TAZ activity (Nouri et al., 2019a).

### NanoLuc-Based YAP-TEAD Biosensor and Small Molecule Screening

Following the development and application of both LATS biosensors, a second NanoBiT system was built to monitor the interaction between YAP and the TEAD family of transcription factors (Nouri et al., 2019b). Repressing the transcriptional output of the Hippo pathway by inhibiting this interaction is a promising avenue for cancer therapy (Liu-Chittenden et al., 2012; Johnson and Halder, 2014; Zhou et al., 2015). Indeed, several peptides and small molecule inhibitors of the YAP/TEAD interaction have been identified as potential therapeutics (Liu-Chittenden et al., 2012; Pobbati et al., 2015; Song et al., 2018). However, none have advanced to clinical trials due to low stability *in vivo*, low cell permeability, or other drawbacks. As such, we developed a YAP-TEAD NanoBiT biosensor to enable high throughput screening for new small molecule inhibitors (Nouri et al., 2019b).

Similar to both iterations of the LATS biosensors, the YAP-TEAD biosensor made use of protein fragments based on structural insights of the YAP-TEAD interface (Li et al., 2010). Of note, extensive analysis was conducted to determine the optimal orientation of this biosensor. There are 8 possible orientations of a YAP-TEAD biosensor if the NanoBiT constituents are cloned onto the N or C-termini of the YAP and TEAD protein fragments (2 proteins—YAP and TEAD, by 2 luciferase constituents—SmBiT and LgBiT, by 2 termini). As such, 8 versions of the YAP-TEAD biosensor representing each of the distinct orientations were cloned. Ultimately, SmBiT-YAP and LgBiT-TEAD (SmBiT and LgBiT linked to the N-termini of YAP and TEAD, respectively) showed the highest luminescent signal. While most versions of the YAP-TEAD biosensor displayed an easily detectable luminescent signal, optimal placement of the luciferase constituents is an important consideration when using split luciferase systems.

After validation, the YAP-TEAD biosensor was used in a large-scale screen (2,688 compounds) for small molecule inhibitors of this interaction (Nouri et al., 2019b). Seventy-one compounds decreased the luminescent activity more than two-fold. Several follow-up screening protocols further refined the list of candidate YAP-TEAD inhibitors. The top hit from these secondary screens, celastrol, was validated as an anti-cancer agent in breast and lung cancer cell lines. Notably, secondary screening that did not make use of the NanoBiT biosensor was an important step in the validation procedures; some false positives were attributed to compounds that exert a non-specific quenching effect on the luciferase component of the biosensor, rather that inhibition of the PPI of interest. Nevertheless, a biosensor-based approach to enable high throughput drug screening proved to be extremely effective.

### BRET and AP-MS to Study Protein–Protein Interaction in the Hippo Pathway

Bioluminescence resonance energy transfer (BRET) is a similar method of studying PPIs (Boute et al., 2002; Pfleger and Eidne, 2006). Like split luciferase systems, BRET relies on protein fusion with two binding partners of an interaction pair. In contrast, BRET involves the transfer of light from a functional luciferase donor to a fluorophore acceptor that emits light of a different wavelength (Figure 2C), rather than complementation of two non-functional luciferase components (Boute et al., 2002; Pfleger and Eidne, 2006). Overall, it is difficult to declare the superiority of one of these approaches over the other; both systems are highly sensitive and give real time information on PPIs.

Promega Corporation has developed a BRET system that also makes use of NanoLuc luciferase (Machleidt et al., 2015). A separate group has created a distinct NanoLuc-based BRET platform, dubbed BRET^*n*^, that uses a different fluorophore (Mo et al., 2016). Following validation of BRET^*n*^, this group applied their system in a high throughput screen for small molecule inhibitors of PRAS40 dimerization. Furthermore, the BRET^*n*^ platform was used to map out a small Hippo pathway interactome by cloning the donor (NanoLuc) and acceptor (a yellow fluorescent protein variant) in various configurations onto established Hippo pathway components (RASSF1, MST1, LATS2, YAP1, TEAD2, and 14-3-3) (Mo et al., 2016). The BRET^*n*^ system correctly identified several established Hippo pathway PPIs and proposed two novel PPIs: LATS2 homodimerization and RASSF1-LATS2 interaction. In essence, a network of biosensor fusion proteins was used to characterize the interactome of several Hippo pathway components.

Initial characterization of the Hippo pathway interactome was accomplished by affinity purification mass spectrometry studies from 3 separate groups in 2013 and 2014 (Couzens et al., 2013; Kwon et al., 2013; Moya and Halder, 2014; Wang et al., 2014). AP-MS relies on the purification and characterization of protein complexes bound to tagged ‘bait’ proteins. These 3 studies used established components of the canonical Hippo pathway as bait proteins to reveal an extensive interactome in both drosophila (Kwon et al., 2013) and humans (Couzens et al., 2013; Wang et al., 2014). Furthermore, Couzens et al. supplemented their AP-MS findings using a biotin labelling approach and investigated changes in the Hippo pathway interactome associated with phosphatase inhibition by okadaic acid (Couzens et al., 2013). Overall, these analyses provide an excellent overview of the Hippo PPI network.

Both AP-MS and split luciferase/BRET based methodologies provide unique insights and hold several key advantages over the other method. In general, AP-MS is better suited to large-scale characterization of interactomes to provide a high-level overview of a PPI network, whereas luciferase complementation or BRET can be used for more extensive study of individual PPIs. Mainly, this is because luciferase methods require molecular cloning or genome editing of both interaction partners, whereas AP-MS only requires tagging of 1 “bait” protein. Therefore, only one or a select few interactions can be studied easily with luciferase methods. While this does not allow for extensive or wholistic characterization of an interactome, a sensitive biosensor screen can be used to discover both direct and indirect upstream regulators of one (or a select few) high interest PPIs (Azad et al., 2018; Nouri et al., 2019a).

The main advantages of split luciferase or BRET methods are operational simplicity, high sensitivity, and the capacity to give real time information in live cells. AP-MS requires substantial expertise and familiarity with the pipeline for analysis, whereas luciferase techniques require only molecular cloning or genome editing and some specialized equipment to yield easily interpretable results. Consequently, split luciferase or BRET techniques are more accessible. In addition, luciferase assays are better suited for thorough characterization of one, high-interest PPI because of their excellent overall sensitivity. Also, the ability to purify fusion protein biosensors is incredibly useful for high throughput drug screening. Finally, many luciferase substrates are permeable to the cell membrane. This allows for *in vivo* study of PPIs in a time and space dependent manner that accounts for cellular compartmentalization.

## Future Directions, Current Limitations, and Conclusions

Both BRET and split luciferase complementation assays are powerful tools to facilitate future study of protein–protein interaction in the Hippo pathway. The recent development of NanoLuc luciferase, demonstrating extremely high sensitivity and improved thermal stability, has enabled the implementation of these systems for increasingly useful applications. Namely, for high throughput screening processes and for sensitive characterization of small protein network interactomes.

On the immediate horizon for future studies is the development of biosensors to monitor the interaction of other Hippo pathway PPIs of interest. For example, the LATS–ITCH interaction is a crucial regulator of LATS stability that plays an important role in proliferation of breast cancer cells, and is therefore an attractive drug target for molecular chemotherapeutics (Ho et al., 2011; Yeung et al., 2013). A NanoBiT-LATS/ITCH biosensor could be used to screen for small molecule inhibitors of this interaction for breast cancer therapy. Since the Hippo pathway also plays important roles in fibrosis, wound healing, and tissue regeneration, inhibition of core Hippo kinase activity may be desirable in the context of regenerative therapies (Moya and Halder, 2019; Dey et al., 2020). In particular, Hippo pathway inhibition to promote YAP/TAZ activity shows significant potential in activating heart repair mechanisms following myocardial damage (Xin et al., 2013; Leach et al., 2017; Zheng et al., 2020). Biosensor screens could also be used to facilitate the discovery of small molecules for this purpose. Also worth consideration, luciferase technologies may prove useful for studying the influence of dynamic mechanical cues on Hippo pathway activity. For example, YAP and TAZ signaling is altered in response to disturbed blood flow (Wang et al., 2016). A YAP/TAZ-TEAD or LATS biosensor could be a useful tool to study the influence of shear stress on Hippo pathway activity in the context of a dynamic, *in vitro* blood flow model, with implications for better characterization of the role of Hippo signaling in vasculature disease.

In addition, luciferase technology has promising applications for *in vivo* imaging of molecular processes. Recently, a firefly based split luciferase complementation assay was used to monitor GPCR signaling *in vivo* (Kono et al., 2017). Luciferase activity was responsive to inhibitors of the interaction being studied, meaning that split luciferase assays could potentially be used to assess the efficacy of targeted molecular therapeutics in pre-clinical animal models. Notably, the NanoBiT complementation system is not suitable for *in vivo* imaging due to its short emission wavelength of approximately 460 nm (Hall et al., 2012). This is where BRET based systems are preferred; many fluorophores have been engineered to fluoresce at a more red-shifted emission spectrum, which is better for penetrating tissue (Yuan et al., 2013). Hiblot et al. have recently developed a system of NanoLuc-BRET biosensors with a range of emission maxima from 480 to 680nm which could perhaps be applied for *in vivo* imaging (Hiblot et al., 2017).

Historically, the limitations of luciferase technologies have been low stability and large size. However, NanoBiT technology presents significant improvements in both of these areas while also showing improved sensitivity (England et al., 2016). Other than a low emission spectrum that is not optimal for *in vivo* imaging, the primary limitation of NanoBiT is its current cost. Furimazine, the NanoLuc substrate, is not generically available and therefore the cost of the NanoLuc platform is higher than other luciferase systems. Perhaps the discovery of new NanoLuc substrates will decrease the cost in the future. In addition, an important generic consideration for split luciferase assays is the requirement for fusion protein generation, introducing the potential for alteration of protein function or steric inhibition of the endogenous PPI. This limitation can be mitigated by using a small luciferase and by testing of different biosensor orientations. Furthermore, new luciferases or continued engineering of NanoLuc could potentially yield an even smaller enzyme.

In summary, this review presents an exciting new approach to studying protein–protein interaction in the Hippo pathway using luciferase-based biosensors. Namely, the main advantages of these systems are operational simplicity, high sensitivity, and the capacity to transmit reliable information in real time. These qualities lend themselves extremely well to high throughput screening processes, both for upstream kinase regulators and for targeted molecular therapeutics. Beyond their applications for screening, luciferase-based biosensors are convenient and easy to use. Future studies could look to apply this approach for expanded screens with novel biosensors, study of mechanical transduction, or for *in vivo* imaging.

## Author Contributions

AP wrote the manuscript. XY revised the manuscript. Both authors contributed to the article and approved the submitted version.

## Conflict of Interest

The authors declare that the research was conducted in the absence of any commercial or financial relationships that could be construed as a potential conflict of interest.

## Abbreviations

AP-MS: affinity purification mass spectrometry
BRET: bioluminescence resonance energy transfer
BRET^*n*^: BRET platform developed by Mo et al. that uses NanoLuc as a luciferase donor
CLuc: C-terminal segment of firefly luciferase in a split luciferase system
FLuc: firefly luciferase
LgBiT: large luciferase constituent of NanoLuc for use in split luciferase complementation system
NanoBiT: NanoLuc binary technology i.e., a split luciferase system derived from NanoLuc
NanoLuc: a luciferase enzyme derived from *Oplophorus gracilirostris*
NLuc: N-terminal segment of firefly luciferase in a split luciferase system
PPI: protein–protein interaction
SmBiT: Small luciferase constituent of NanoLuc for use in s split luciferase complementation system
YAP15: 15 amino acid segment of the YAP1 protein containing the consensus LATS phosphorylation site at serine-127.
